# Muscle Ultrasound Echo Intensity Detects Insulin Resistance Before Changes in HbA_1c_ or Fasting Glucose, Independent of BMI: A Novel Tool for Early Risk Stratification

**DOI:** 10.1002/jum.70126

**Published:** 2025-11-18

**Authors:** Steven B. Soliman, Jacob E. Leuteneker, Olivia K. Chugh, Tao Zhang, Becca Tuska, Thomas Rode, Maria J. Sanchez, Eric D. Buras, Jeffrey F. Horowitz

**Affiliations:** ^1^ Division of Musculoskeletal Radiology, Department of Radiology University of Michigan Ann Arbor Michigan USA; ^2^ Substrate Metabolism Laboratory, School of Kinesiology University of Michigan Ann Arbor Michigan USA; ^3^ Pediatric Department PedCare Medical Group Dallas Texas USA; ^4^ Division of Metabolism, Endocrinology, and Diabetes, Department of Internal Medicine University of Michigan Ann Arbor Michigan USA

**Keywords:** biomarker, glycemic control, insulin resistance, obesity, skeletal muscle echo intensity, ultrasound

## Abstract

**Objectives:**

To quantify the association between skeletal muscle echo intensity (MEI), measured by ultrasound, and clinical markers of insulin resistance and glycemic control, and evaluate MEI's diagnostic accuracy in identifying insulin resistance.

**Methods:**

In this cross‐sectional study, 20 adults with obesity (mean body mass index [BMI] 34.4 ± 2.6 kg/m^2^, mean age 33 years, 40% female) and 8 healthy, lean adults (mean BMI 22.5 ± 1.4 kg/m^2^, mean age 25 years, 75% female), all without diabetes or metabolic disease, underwent laboratory testing (HbA_1c_, 2‐hour oral glucose tolerance testing with insulin for Matsuda Index), muscle ultrasound (deltoid, vastus lateralis), and DEXA for sarcopenia indices. Two blinded research assistants independently analyzed 336 ultrasound images to quantify MEI.

**Results:**

Increased MEI was significantly associated with greater insulin resistance (lower Matsuda Index; *r* = −.47, *p* = .011), particularly in women (*r* = −.56, *p* = .039). MEI z‐scores identified insulin resistance with an AUROC of 0.872 (95% CI 0.742–1.000). At the optimal threshold of z = 1.96, sensitivity was 94.4%, specificity 80%, accuracy 89.3%, and Youden's index 0.744. MEI accurately identified insulin resistance despite normal HbA_1c_ and fasting glucose. Increased MEI in both muscles suggested global skeletal muscle changes. Among participants with obesity, MEI did not correlate with BMI but was negatively correlated with sarcopenia indices (*r* = −.56, *p* = .0096) and body weight (*r* = −.50, *p* = .0233).

**Conclusions:**

MEI is an accurate, noninvasive biomarker for insulin resistance and may detect muscle alterations before conventional markers emerge. Its independence from BMI and conventional markers supports MEI's use in early risk stratification and identification of individuals at risk for metabolic dysfunction who might otherwise go undetected.

**Abbreviations**
ASMI: appendicular skeletal muscle index
ASMI: appendicular skeletal muscle index
AUROC: area under the ROC curve
BMI: body mass index
DEXA: dual‐energy X‐ray absorptiometry
FFM: fat‐free mass
FFMI: fat‐free mass index
ICC: intraclass correlation coefficient
MEI: muscle echo intensity
OGTT: oral glucose tolerance test
ROC: receiver operating characteristic

Insulin resistance is a primary defect underlying the development of type 2 diabetes mellitus (T2D), often preceding β‐cell failure and overt systemic metabolic dysfunction by decades.^1^, ^2^, ^3^ Both insulin resistance and T2D are closely tied to obesity,^4^, ^5^, ^6^, ^7^ which now affects more than 40% of adults in the United States.^8^, ^9^ Despite the use of current screening methods such as hemoglobin A_1c_ (HbA_1c_) and fasting glucose, and even with the United States Preventive Services Task Force's updated guidelines,^10^ hundreds of millions of individuals worldwide, including one‐third of the United States population, remain undiagnosed with T2D or prediabetes, indicating a silent epidemic of metabolic dysfunction.^11^, ^12^, ^13^, ^14^, ^15^ Critically, there is still no simple, noninvasive, and inexpensive method for early identification of developing insulin resistance in at‐risk individuals. Alarmingly, at the time of T2D diagnosis, approximately half of individuals have already developed 1 or more irreversible complications, reflecting the clinical consequences of this silent epidemic. Early detection of metabolic dysfunction is critical for timely intervention and the prevention of adverse and irreversible outcomes.^11^, ^12^, ^13^, ^14^, ^15^

Skeletal muscle is central to systemic glucose metabolism and plays a key role in insulin sensitivity.^1^, ^16^, ^17^, ^18^, ^19^, ^20^, ^21^, ^22^, ^23^, ^24^ Our prior research demonstrated that elevated skeletal muscle echo intensity (MEI), as measured by ultrasound, could accurately identify individuals with T2D and prediabetes, independent of age or disease duration.^12^, ^13^, ^14^ Notably, increased MEI was observed even in individuals with previously undiagnosed T2D and prediabetes, sometimes before HbA_1c_ levels become abnormal.^13^, ^25^ These findings supported MEI's potential as a simple, noninvasive, and cost‐effective tool for early detection of metabolic dysfunction. However, in earlier work, MEI did not correlate with insulin sensitivity as measured by the gold standard hyperinsulinemic‐euglycemic clamp‐derived M values.^25^ This raised important questions: Was the lack of correlation due to limited sample size, or does MEI primarily reflect the presence of insulin resistance rather than its severity?

Building on these findings, the current study aimed to address these gaps by quantitatively assessing the association between skeletal MEI and clinical markers of insulin resistance and glycemic control in adults with and without obesity. Additionally, we evaluated MEI's diagnostic accuracy in identifying insulin resistance. We hypothesized that increased MEI would serve as an accurate, noninvasive, and accessible biomarker for insulin resistance, potentially identifying muscle changes before elevations in HbA_1c_ and fasting glucose. By clarifying these relationships, this study seeks to demonstrate MEI's role as a tool for early risk stratification and to guide future approaches for timely intervention.

## Materials and Methods

This study was performed in accordance with the ethical standards of our institutional research committee and with the 1964 Declaration of Helsinki and its later amendments or comparable ethical standards. The University of Michigan Institutional Review Board approval was obtained for this prospective study (HUM00242823), and written informed consent was obtained. Our study complied with the Health Insurance Portability and Accountability Act.

### Study Subjects

Between August 2023 and July 2025, we prospectively enrolled 20 subjects with obesity (body mass index [BMI] ≥30–≤40 kg/m^2^) who had no history of diabetes or other metabolic disease. For comparison, a group of 8 healthy, lean individuals (BMI ≥18.5–≤24.9 kg/m^2^) was recruited using the same inclusion and exclusion criteria.

Inclusion criteria included adult men and women aged 18–40 years of any race or ethnicity. To minimize the effects of hormonal fluctuations on muscle composition, only premenopausal women with regular menstrual cycles were included. Women who were pregnant or breastfeeding were excluded.^26^

Participants were excluded if they had any relevant medical conditions or diagnoses in the previous year that could impact the sonographic appearance of muscle, such as muscle injury, paralysis, myositis, rhabdomyolysis, or other myopathies.^12^, ^13^, ^14^, ^25^ Additionally, individuals taking medications known to alter lipid or glucose metabolism or inflammation were not eligible, to control for potential confounding effects from these agents. To standardize conditions, all subjects fasted for 12 hours before their study visit to control for hydration and dietary variability.^12^, ^13^, ^25^

### Ultrasound of Skeletal Muscles

For both the obese and lean groups, ultrasound examinations of the dominant‐side deltoid and vastus lateralis muscles were conducted by 1 musculoskeletal radiologist (15 years' experience). Imaging was performed at standardized anatomical locations: the deltoid muscle was assessed at the anterolateral shoulder, specifically one‐fifth of the distance from the acromion to the lateral epicondyle, while the vastus lateralis muscle was scanned at the anterolateral mid‐thigh, positioned halfway between the greater trochanter and the patella. Prior to imaging, participants rested in a supine and relaxed state to mitigate variability due to muscle contraction.^25^, ^27^ To ensure consistency, light transducer pressure was applied, and all scans were taken at a perpendicular angle to the underlying bone or fascia, focusing on areas where these structures appeared most reflective (ie, bone or fascia appear brightest). Each muscle was imaged in both the short‐ and long‐axes, with 3 consecutive images recorded per axis. This protocol yielded a total of 12 images per participant, resulting in 336 images across all 28 subjects. Ultrasound scans were obtained using a standardized protocol with a 12‐MHz linear transducer (ARIETTA 850; FUJIFILM Medical Co., Ltd., Tokyo, Japan), preset to B‐mode, 60 decibel gain, 70 decibel dynamic range, 5 cm depth, and automated dynamic focusing to optimize image clarity across the entire field without manual focal zone adjustment.

### Laboratory Analyses (HbA_1c_ , 2‐Hour Oral Glucose Tolerance Test with Insulin Measures)

After completing the ultrasound examinations, all participants underwent blood testing, including HbA_1c_ and a 2‐hour oral glucose tolerance test (OGTT) with insulin levels measured throughout the procedure. For the OGTT, an intravenous catheter was placed in a forearm vein, and a baseline blood sample was drawn. Participants then ingested a 75‐gram glucose solution (Fisherbrand), and additional blood samples were collected every 15 minutes over 2 hours while seated. Plasma and serum samples were stored at −80°C until analysis of glucose and insulin concentrations.

Insulin sensitivity was assessed using the Matsuda Index, which is calculated using the formula:

$$\text{Matsuda Index}=\frac{10,000}{\sqrt{\left(\text{fasting glucose}\times \text{fasting insulin}\right)\times \left(\text{mean glucose}\times \text{mean insulin}\right)}}$$

This index incorporates both fasting and postprandial glucose and insulin levels (0, 30, 60, 90, and 120 minutes), providing a comprehensive measure of peripheral (skeletal muscle) and hepatic insulin sensitivity.^2^, ^28^, ^29^, ^30^ A Matsuda Index less than 4 was used to define insulin resistance.^2^, ^30^, ^31^, ^32^, ^33^ Although the hyperinsulinemic‐euglycemic clamp is considered the gold standard for assessing insulin resistance,^2^, ^25^, ^34^, ^35^ we used the Matsuda Index due to its strong correlation with clamp‐derived measures, lower invasiveness, and greater practicality for clinical research.^28^, ^29^, ^36^

### Dual‐Energy X‐Ray Absorptiometry (DEXA) Scans

All 28 participants underwent body composition assessments using DEXA with a GE‐Lunar iDXA scanner. Measurements included BMI and percentage of body fat. The scans also provided data on fat‐free mass (FFM) and appendicular lean mass, which were subsequently used to calculate sarcopenia indices including appendicular skeletal muscle index (ASMI) and FFM index (FFMI).^25^, ^37^, ^38^

### Ultrasound MEI Quantifications

A total of 336 ultrasound images from the deltoid and vastus lateralis muscles were independently evaluated by 2 trained research assistants to ensure objectivity and reliability. Both reviewers were blinded to participant group, clinical measures, BMI, and demographic information.

MEI was quantitatively assessed using established greyscale analysis methods, where pixel intensity ranges from 0 (black) to 255 (white).^13^, ^25^, ^27^, ^39^, ^40^, ^41^ Each research assistant used ImageJ software (v1.54p, Madison, WI, USA) to analyze the images. For each image, a standardized rectangular region of interest (3 cm × 1.4 cm) was placed centrally within the muscle, carefully avoiding inclusion of bone or fascia. A total of 12 images per participant were analyzed, with each assistant making 3 replicate measurements per image (yielding 36 measurements per subject per assistant). For each image, the mean MEI was determined per assistant, and final MEI values for individual muscles were calculated by averaging the results from both assistants.

For each participant, MEI *z*‐scores for the deltoid and vastus lateralis muscles were calculated using the mean and standard deviation of the healthy, lean group as reference values.^42^, ^43^, ^44^ The *z*‐score was computed using the formula:

$$z=\frac{X-\mu }{\sigma }$$

where *X* is the mean MEI value for each subject, *μ* is the mean MEI of the healthy, lean group, and *σ* is the corresponding standard deviation.

### Statistical Analysis

Between‐group differences (obesity versus healthy, lean) were assessed using Fisher's exact or Mann–Whitney *U* tests, depending on data distribution. Pearson correlation coefficients were calculated to evaluate associations between MEI and clinical/metabolic parameters, including Matsuda Index, fasting glucose, HbA_1c_, skeletal muscle mass indices, and body weight. Subgroup analyses were performed by sex within each group to explore potential sex‐specific associations.

To assess the diagnostic performance of MEI in identifying insulin resistance (defined as Matsuda Index <4),^2^, ^30^, ^31^, ^32^, ^33^ receiver operating characteristic (ROC) curve analysis was performed. Optimal MEI z‐score thresholds were determined using Youden's Index.^14^ Diagnostic metrics, including area under the ROC curve (AUROC), sensitivity, specificity, and overall accuracy, were calculated to evaluate classification performance.

Inter‐rater agreement for mean MEI measurements of the deltoid and vastus muscles was evaluated using the intraclass correlation coefficient (ICC), calculated from 2‐way mixed‐effects models. Interpretation of ICC values was based on Rosner's criteria: 0–0.40 = poor agreement, >0.40–0.75 = good agreement, and >0.75–1.00 = excellent agreement. Statistical analyses were performed using R (version 4.5.1). A *p*‐value of <.05 was considered statistically significant.

## Results

### Participant Characteristics

Characteristics of the study cohorts are presented in Table 1. Consistent with the study design, the obesity group exhibited significantly higher BMI, body weight, and percent body fat compared to the healthy, lean group. Additionally, mean HbA_1c_ and fasting glucose levels were not significantly different between cohorts, consistent with the inclusion of only metabolically healthy individuals without diabetes or other metabolic diseases in the obesity group.

**Table 1:** Demographics and Characteristics of Study Cohorts

	Healthy Lean Subjects	Subjects with Obesity	*p*‐Value^a^
	(n = 8)	(n = 20)	
Sex			
Female	6 (75%)	8 (40%)	.209
Male	2 (25%)	12 (60%)	
Race			
White or Caucasian	4 (50%)	15 (75%)	.372
Asian	4 (50%)	3 (15%)	
Black or African American	0 (0%)	2 (10%)	
Ethnicity			
Non‐Hispanic	6 (75%)	18 (90%)	.555
Hispanic	1 (12%)	1 (5%)	
Other or unknown	1 (12%)	1 (5%)	
Age (years)	25.0 ± 6.9	33.0 ± 5.8	.026
Height (cm)	170.0 ± 9.8	172.7 ± 10.2	.508
Weight (kg)	64.8 ± 5.0	103.0 ± 14.6	<.0001
HbA_1c_	5.2 ± 0.2	5.6 ± 1.2	.419
Fasting glucose	84.2 ± 10.0	86.2 ± 17.2	.690
Body mass index (kg/m^2^)	22.5 ± 1.4	34.4 ± 2.6	.0001
Body fat (%)	30.4 ± 8.2	42.4 ± 5.2	.0003

Categorical data are represented as frequency (percentage of column). Numerical data are represented as mean ± standard deviation.

^a^ *p*‐Values for comparing healthy, lean and obese cohorts: Fisher's exact test for categorical variables; Mann–Whitney *U* test for continuous variables.

### Matsuda Index

**Figure 1:**
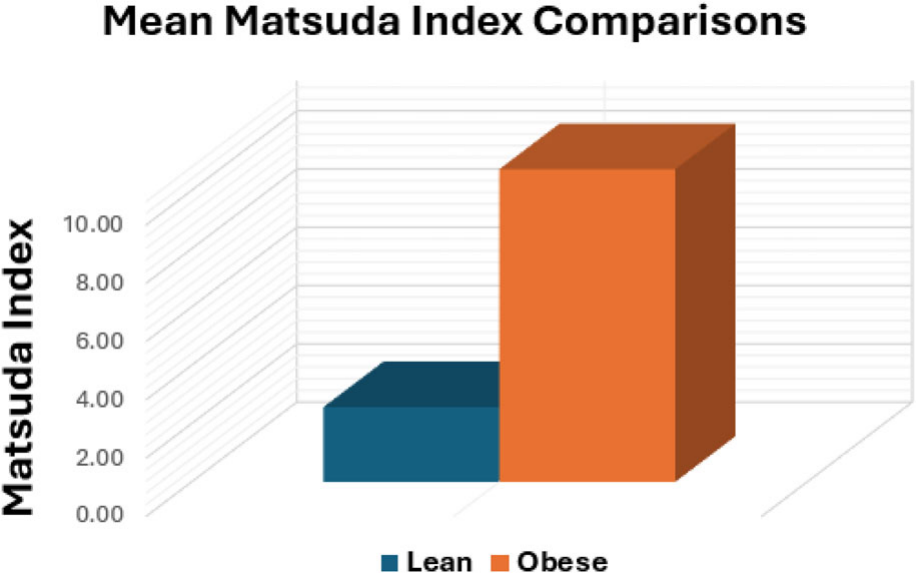
Comparison of mean Matsuda Index values between lean and obese cohorts.

The mean Matsuda Index was 2.57 ± 1.10 in the obesity group and 10.73 ± 7.27 in the healthy, lean group (Figure 1), representing a >75% lower value in the obesity group (*p* = .0081), consistent with reduced insulin sensitivity. Eighteen of the 20 subjects with obesity had a Matsuda Index <4, indicating insulin resistance; the remaining 2 had values of 4.19 and 5.09, consistent with borderline insulin resistance.

### Skeletal MEI

**Figure 2:**
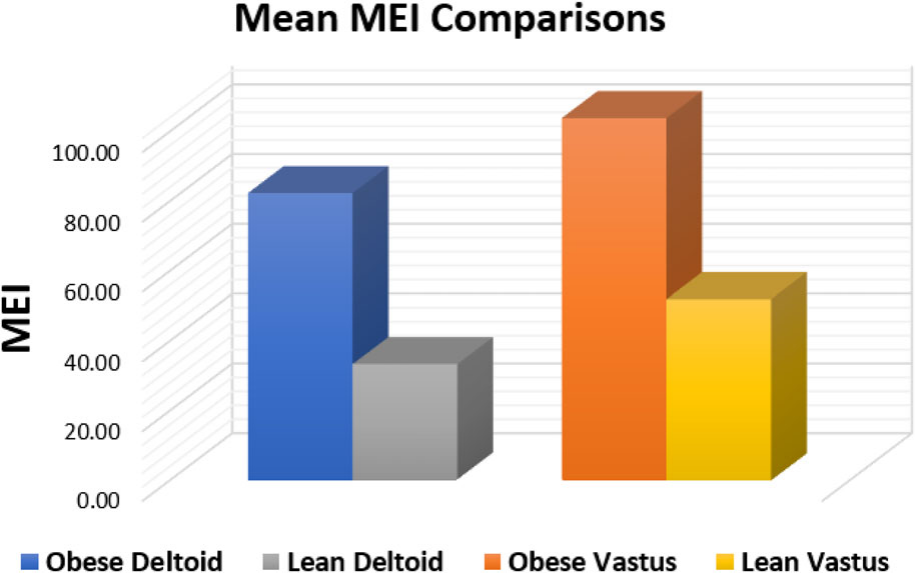
Comparison of mean skeletal MEI measurements in the deltoid and vastus lateralis muscles between obese and healthy, lean cohorts.

There was a highly significant difference in mean deltoid MEI values between the obesity group (82.39 ± 28.32) and the healthy, lean group (33.41 ± 6.21) (*p* < .001), with a mean difference of 48.98, representing a 146.6% increase relative to the lean group (Figure 2). Similarly, mean vastus lateralis MEI values were significantly higher in the obesity group (103.91 ± 29.96) compared to the lean group (51.97 ± 12.83) (*p* < .001), with a mean difference of 51.94, corresponding to a 99.9% increase (Figure 2). Effect sizes were very large (Cohen's *d* = 2.01 for deltoid and *d* = 1.96 for vastus lateralis), indicating strong between‐group differences.

### Interobserver Agreement

There was excellent interobserver agreement in MEI measurements between the 2 blinded research assistants for both the deltoid (ICC = 0.996; 95% CI: 0.992–0.998) and vastus lateralis (ICC = 0.999; 95% CI: 0.998–1.000) muscles.

### Relationships between MEI and Measures of Insulin Sensitivity and Glycemic Control

**Figure 3:**
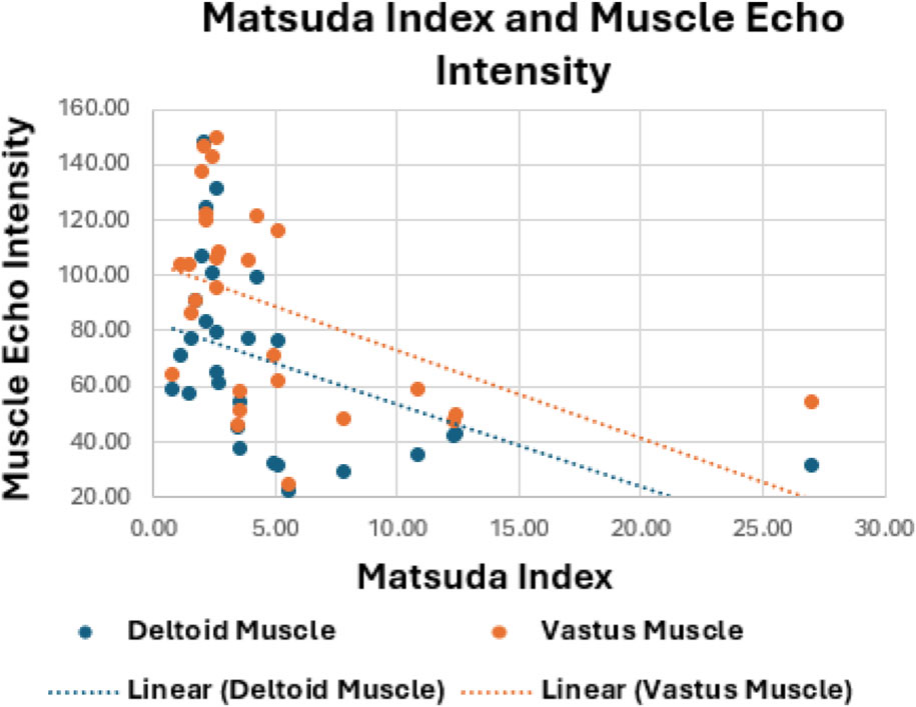
Scatter plot showing that increased skeletal MEI is significantly associated with greater insulin resistance, as reflected by lower Matsuda Index values in both the deltoid (*r* = −.47, *p* = .011) and vastus lateralis muscles (*r* = −.47, *p* = .011).

Increased MEI was significantly associated with greater insulin resistance, as reflected by lower Matsuda Index values in both the deltoid (*r* = −.47, *p* = .011) and vastus lateralis muscles (*r* = −.47, *p* = .011) (Figure 3). This association was most pronounced in women (deltoid, *r* = −.54, *p* = .046; and vastus, *r* = −.56, *p* = .039). Increased MEI in both upper and lower extremity muscles suggests global skeletal muscle changes.

**Figure 4:**
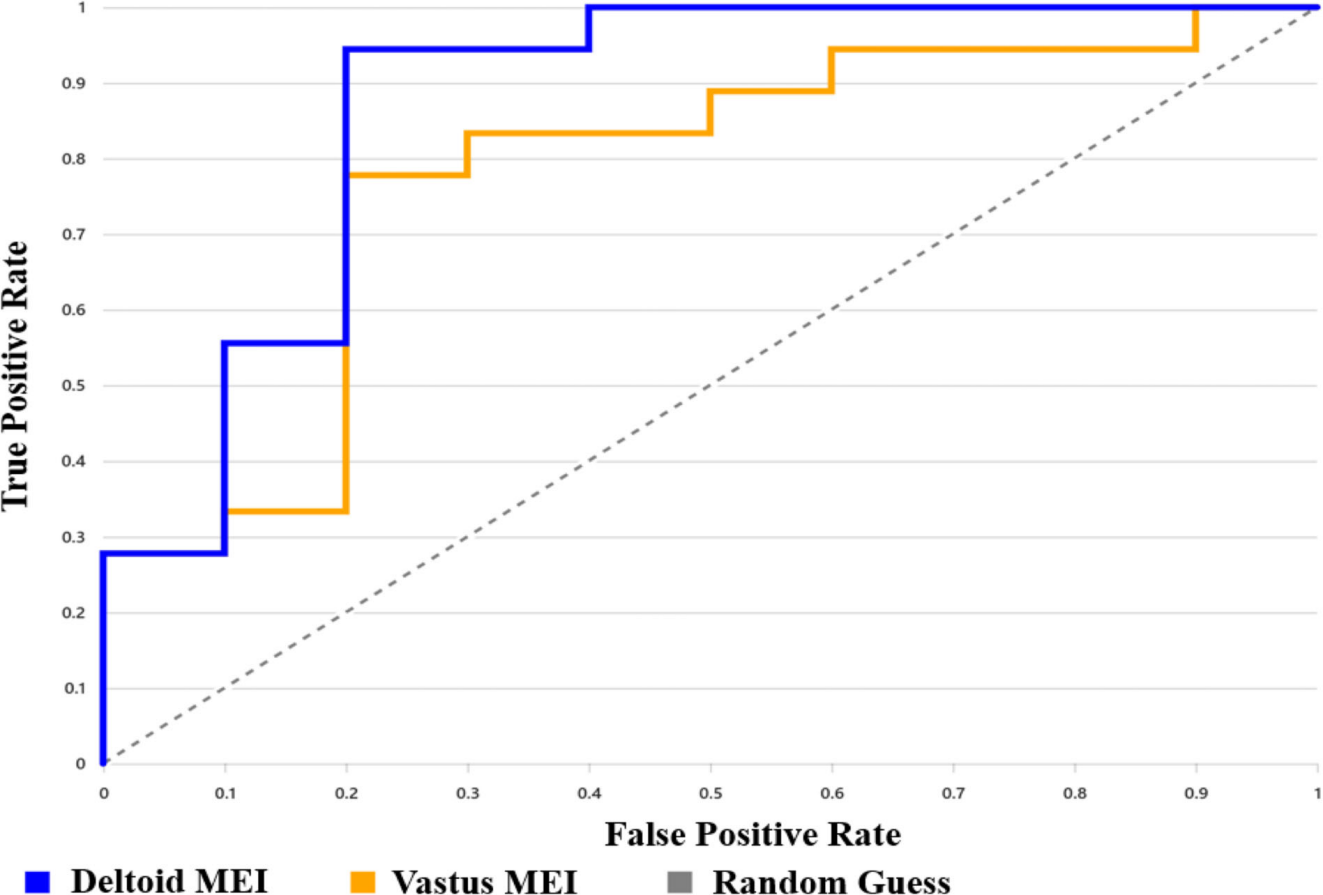
AUROC analysis of deltoid and vastus lateralis MEI z‐scores for identifying insulin resistance. Deltoid MEI z‐scores yielded an AUROC of 0.872 (95% CI: 0.742–1.000), with sensitivity of 94.4%, specificity of 80%, and overall accuracy of 89.3%. Vastus lateralis MEI z‐scores yielded an AUROC of 0.778 (95% CI: 0.606–0.95), with sensitivity of 77.8%, specificity of 80%, and accuracy of 78.6%.

Deltoid MEI z‐scores accurately identified insulin resistance, with an AUROC of 0.872 (95% CI: 0.742–1.000). At the optimal threshold of z = 1.96 (determined by Youden's index), sensitivity was 94.4%, specificity was 80%, accuracy was 89.3%, and the maximum Youden's index was 0.744 (Figure 4).

Vastus lateralis MEI z‐scores were also a useful biomarker for insulin resistance, with an AUROC of 0.778 (95% CI 0.606–0.95). At the optimal threshold of *z* = 2.66, sensitivity was 77.8%, specificity was 80%, and accuracy was 78.6%, and the maximum Youden's index was 0.578 (Figure 4).

### MEI as an Early Indicator of Insulin Resistance

As noted above, both cohorts exhibited normal HbA_1c_ and fasting glucose levels, with no statistically significant differences in mean values between groups (Table 1). Importantly, MEI values did not correlate with HbA_1c_ or fasting glucose among the 28 subjects without diabetes or metabolic disease. These findings suggest that elevated MEI may precede detectable changes in conventional metabolic markers such as HbA_1c_ or fasting glucose.

### Skeletal MEI, BMI, and Body Weight

In the obesity group, MEI did not correlate with BMI (*r* = −.003, *p* = .9881) but showed a significant negative correlation with body weight in both the deltoid (*r* = −.48, *p* = .0306) and vastus lateralis (*r* = −.50, *p* = .0233) muscles. These findings suggest that MEI reflects muscle alterations related to insulin resistance that are not explained by overall body size, highlighting its potential as a unique imaging biomarker for early risk stratification and identification of individuals at risk for T2D.

### Skeletal MEI and Muscle Mass

In the cohort with obesity, MEI demonstrated a strong negative correlation with muscle mass, as measured by sarcopenia indices. Higher MEI values were significantly associated with lower ASMI and FFMI. Specifically, ASMI was negatively correlated with deltoid MEI (*r* = −.50, *p* = .0231) and vastus lateralis MEI (*r* = −.53, *p* = .0158), while FFMI showed similar negative correlations with deltoid MEI (*r* = −.56, *p* = .0110) and vastus lateralis MEI (*r* = −.56, *p* = .0096). These findings suggest that elevated MEI may reflect reduced relative muscle mass in the context of obesity and insulin resistance, conditions known to be closely linked.^1^, ^16^, ^17^, ^18^, ^19^, ^20^, ^21^, ^22^, ^23^, ^24^

## Discussion

This study is the first to establish a significant association between MEI and insulin resistance, with high diagnostic accuracy. These findings highlight MEI's potential as a simple, noninvasive, and cost‐effective biomarker for the early detection of metabolic dysfunction. Notably, MEI did not correlate with BMI or conventional biomarkers such as HbA_1c_ or fasting glucose, underscoring its value as a unique and independent biomarker. This independence from traditional markers and body size suggests that MEI may detect muscle alterations that precede abnormalities identified by current screening methods, offering a novel window into early metabolic changes.

MEI may therefore serve as an early indicator of insulin resistance, particularly in individuals who appear metabolically healthy by traditional criteria. The ability to detect metabolic dysfunction before conventional markers become abnormal could transform preventive care, address the silent epidemic of undiagnosed metabolic disease, and reduce the burden of diabetes‐related complications. These findings build on prior research and emphasize MEI's potential to fill critical gaps in current screening strategies.

Our previous work has shown that increased MEI can accurately detect both T2D and prediabetes, and in some cases, identify affected individuals before HbA_1c_ levels become elevated.^12^, ^13^, ^14^ These findings are supported by additional studies demonstrating MEI's utility in detecting metabolic dysfunction.^25^, ^45^, ^46^ This is particularly important given that globally, nearly half of the 589 million individuals with T2D and over 80% of the 635 million with prediabetes remain undiagnosed. This diagnostic gap disproportionately affects underserved and underrepresented populations, contributing to increased mortality and reduced life expectancy. This widespread underdiagnosis reflects a silent epidemic of metabolic dysfunction that progresses unnoticed until irreversible complications emerge. T2D is often asymptomatic in its early stages, and by the time it is diagnosed, approximately 50% of patients have already developed irreversible complications. This contributes to a staggering global economic burden exceeding $1 trillion annually, including $413 billion in the United States alone. Yet, early detection and intervention can reduce the risk of complications by up to 40%, and proactive management of prediabetes can prevent progression to T2D in more than half of cases, significantly improving both life expectancy and quality of life.^11^, ^12^, ^13^, ^14^, ^15^, ^25^

Importantly, insulin resistance is recognized as a primary defect in the pathogenesis of T2D, often preceding overt metabolic dysfunction by decades.^1^, ^2^, ^3^ Despite its clinical importance, insulin resistance is not routinely screened due to the complexity, invasiveness, and cost of current assessment methods.^25^, ^34^, ^35^ Currently, no simple, noninvasive, accurate, and cost‐effective method exists for early detection of metabolic dysfunction, representing a critical gap in both clinical care and public health systems.

Given the increasing availability of low‐cost, handheld point‐of‐care ultrasound devices, and the potential for AI‐driven MEI analysis,^14^ our findings suggest that early detection of insulin resistance could be achieved by frontline practitioners with minimal training.^12^, ^13^, ^14^, ^25^, ^47^, ^48^, ^49^, ^50^ Moreover, our ongoing efforts to integrate AI/machine learning aim to automate MEI analysis, reduce operator dependency, and improve accessibility and scalability across clinical settings. Muscle ultrasound thus holds promise as a low‐cost, accurate, and noninvasive tool for early identification of insulin resistance. This approach could have tremendous clinical impact, enabling timely interventions to prevent the development of T2D. These findings lay the foundation for future studies to validate MEI across larger and more diverse populations, and to explore its integration into routine clinical workflows for early metabolic risk assessment and prevention.

### Limitations and Histologic/Mechanistic Insights

Several limitations of this study should be acknowledged. First, the cohort size was relatively small, primarily due to the time‐intensive nature of the protocol, associated costs, and logistical challenges in recruitment and scheduling. Active enrollment is ongoing to expand the study population. Second, we did not conduct long‐term follow‐up of participants with obesity and insulin resistance to determine progression to prediabetes or T2D. Plans are underway to initiate a longitudinal study to address this gap. Third, the absence of histological correlation limits our ability to fully elucidate the mechanisms underlying increased MEI in obesity and insulin resistance. However, preliminary histological data from the lean and obesity cohorts suggest that elevated MEI reflects early muscle changes, including lipid accumulation and fibrosis, that resemble aging‐related degeneration and may contribute to muscle weakness, even in younger individuals.^1^, ^2^, ^16^, ^17^, ^18^, ^19^, ^20^, ^21^, ^22^, ^23^, ^24^, ^25^, ^40^, ^41^, ^51^, ^52^, ^53^, ^54^, ^55^, ^56^, ^57^, ^58^

Another limitation is the lack of pediatric participants. Given the ongoing epidemic of childhood obesity and the rising prevalence of metabolic disease in youth, this is a critical area for future research.^59^, ^60^, ^61^, ^62^, ^63^, ^64^, ^65^, ^66^ A recent systematic review by Noubiap et al estimated that in 2020, metabolic syndrome affected nearly 3% of children aged 6–12 and 5% of adolescents aged 13–18, equivalent to approximately 26 million children and 36 million adolescents globally.^67^ Early‐life prevention strategies are becoming increasingly critical and if validated in pediatric populations, MEI could serve as a valuable noninvasive biomarker for early detection and intervention, potentially implementable by general pediatricians.

## Conclusion

This study highlights the potential of ultrasound‐based MEI as a novel, accurate, noninvasive, and accessible biomarker for identifying insulin resistance and early metabolic dysfunction, particularly in women. MEI appears capable of accurately detecting skeletal muscle alterations that precede changes in conventional metabolic markers such as HbA_1c_ and fasting glucose, offering a promising avenue for earlier detection and intervention. With further validation, MEI could inform future strategies for risk stratification and enable timely, targeted treatment of metabolic and muscle dysfunction, potentially before irreversible complications arise. Its accessibility, independence from body size, and suitability for point‐of‐care use make it especially valuable for broad clinical application, including in underserved populations where early detection is critical.

## Data Availability

Data sharing not applicable to this article as no datasets were generated or analyzed during the current study.
